# Renal Expression of Light Chain Binding Proteins

**DOI:** 10.3389/fmed.2020.609582

**Published:** 2021-01-13

**Authors:** Thomas Reiter, Sahra Pajenda, David O'Connell, Ciara Lynch, Sebastian Kapps, Hermine Agis, Alice Schmidt, Ludwig Wagner, Nelson Leung, Wolfgang Winnicki

**Affiliations:** ^1^Department of Medicine III, Division of Nephrology and Dialysis, Medical University of Vienna, Vienna, Austria; ^2^School of Biomolecular & Biomedical Science, University College Dublin, Dublin, Ireland; ^3^BiOrbic Bioeconomy Research Centre, University College Dublin, Dublin, Ireland; ^4^Department of Medicine I, Division of Oncology, Medical University of Vienna, Vienna, Austria; ^5^Division of Nephrology and Hypertension, Division of Hematology, Mayo Clinic Rochester, Rochester, MN, United States

**Keywords:** amyloidosis, multiple myeloma, light chains, light chain associated kidney disorders, monoclonal gammopathy, protein micro array analysis

## Abstract

Overproduction of human light chains (LCs) and immunoglobulins can result in various forms of renal disease such as cast nephropathy, monoclonal immunoglobulin deposition disease, LC proximal tubulopathy, AL amyloidosis, and crystal storing histiocytosis. This is caused by cellular uptake of LCs and overwhelmed intracellular transport and degradation in patients with high urine LC concentrations. LC kappa and lambda purification was evaluated by sodium dodecyl sulfate gel electrophoresis. LC and myeloma protein binding to immobilized renal proteins was measured by enzyme-linked immunosorbent assay (ELISA). The human protein microarray (HuProt™) was screened with purified kappa and lambda LC. Identified LC partners were subsequently analyzed *in silico* for renal expression sites using protein databases, Human Protein Atlas, UniProt, and Bgee. Binding of urinary LCs and immunoglobulins to immobilized whole renal proteins from 22 patients with myeloma or plasma cell dyscrasia was shown by ELISA. Forty lambda and 23 kappa interaction partners were identified from HuProt™ array screens, of which 21 were shared interactors. Among the total of 42 interactors, 12 represented cell surface proteins. Lambda binding signals were approximately 40% higher than kappa signals. LC interaction with renal cells and disease-causing pathologies are more complex than previously thought. It involves an extended spectrum of proteins expressed throughout the nephron, and their identification has been enabled by recently developed methods of protein analysis such as protein microarray screening. Further biochemical studies on interacting proteins are warranted to elucidate their clinical relevance.

## Introduction

The most frequent multiple myeloma–associated renal lesion is cast nephropathy (1, 2). This pathological entity is assumed to develop by precipitation of monoclonal free light chains (LCs) associated with uromodulin (3, 4). Cast nephropathy can also be associated with other renal lesions such as monoclonal immunoglobulin deposition disease, LC proximal tubulopathy, AL amyloidosis, and crystal storing histiocytosis (2). Thereby all parts of the nephron can be affected (5). The topology of manifestation is specific to the type and physicochemical properties of the secreted paraprotein (6). In addition to an increased presence of LCs in urine, other factors such as a reduction in tubular flow or an increase in urine salt concentration and the intake of non-steroidal anti-inflammatory drugs (7) can negatively influence cast formation. Pharmacotherapeutic attempts have been performed to improve the outcome in cast nephropathy through modulation of urine pH (8). Also, an approach with intravenous application of a cyclic peptide to inhibit LC aggregation showed promising results in animals (9).

It is well-documented that in healthy individuals LCs appear in primary urine, but are reabsorbed by the proximal tubule through cubilin and megalin according to earlier experiments (10, 11). When the clonal disorder progresses with increasing monoclonal LC production, the reabsorption rate in the proximal tubule is overwhelmed, causing high urine concentration in the distal tubule and LC aggregation and precipitation. The impairment of urine flow in the tubule is not the only issue. Moreover, there is an interaction with tubular cell surface proteins and LCs. This protein binding induces an altered protein expression (12) and an inflammatory reaction leading to interstitial inflammation and consequently to a cast nephropathy-associated interstitial nephritis (13).

Several research reports documented that cast formation can also occur through other mechanisms than monoclonal LCs such as by the antibiotic vancomycin (14) and high concentration of bile salts (15). In this work, we concentrate on LCs, and it is of note that in particular the lambda LC tends to form oligomers (16). LCs from their nature should associate with heavy chains to form immunoglobulins. From this line of thought, it is evident that LCs, when present in high concentration, find various interaction partners even with cell surface proteins of tubular cells. Motivated by the work of previous authors, we immobilized whole renal cell protein lysate on enzyme-linked immunosorbent assay (ELISA) plates and investigated binding intensities of urinary excreted LCs/immunoglobulins obtained from patients with multiple myeloma and controls. To obtain more detailed data on specific binding partners, we purified urinary monoclonal LC kappa and lambda and sought for ways to investigate their interaction potential with renal tubular cell proteins. For this purpose, protein microarrays with 23,000 proteins originating from 16,000 human genes were screened with either purified kappa or lambda LCs. In addition, protein databases, the Human Protein Atlas, UniProt, and Bgee were studied to verify the primary structure and to identify the site and extent of expression in the renal tissue and nephron.

## Materials and Methods

Urine samples from 22 patients treated at the hematology outpatient unit for multiple myeloma or plasma cell dyscrasia were available for analysis in this study. In addition, urine samples from four patients with acute kidney injury due to delayed graft function after renal transplantation and from two healthy individuals were used as controls. Urine obtained from two patients with monoclonal LC excretion and cast nephropathy was chosen for LC purification. The study was approved by the ethics committee of the Medical University of Vienna (EK 2193/2015). All patients were adults older than 18 years and provided written informed consent.

### Patient Characteristics

Patient characteristics including hematological classification and renal histology are listed in Table 1. The two patients with monoclonal LC excretion and cast nephropathy selected for LC purification had the following histological results.

**Table 1:** Demographics of patients with monoclonal gammopathy and paraproteins.

**ID**	**Age**	**Gender**	**LC**	**sCr**	**U[P/C]**	**Hematological classification**	**Renal histology**	**Disease duration**
1	78	m	λ	1.03	49	MM	n.a.	10
2	71	f	κ	1.07	194	MGUS	n.a.	5
3	82	f	κ	0.90	256	MM	n.a.	3
4	81	f	λ	0.97	659	AL amyloidosis	n.a.	3
5	88	f	κ	1.15	1	MGRS	n.a.	6
6	50	m	κ	1.34	214	MM	n.a.	1
7	69	m	κ	0.99	115	MGUS	n.a.	7
8	81	f	λ	0.72	58	MM	n.a.	10
9	70	f	λ	1.11	112	MGRS	LCPT	1
10	77	f	λ	0.64	81	MM	n.a.	10
11	60	m	λ	1.07	115	MM	n.a.	2
12	79	m	λ	2.69	460	MGRS	FGN	2
13	64	f	κ	0.90	274	MM	n.a.	6
14	75	m	λ	0.79	245	MM	n.a.	6
15	84	m	κ	1.94	283	MM	n.a.	9
16	69	m	λ	0.66	1	MGUS	n.a.	6
17	81	m	κ	0.86	118	MM	n.a.	1
18	79	m	λ	1.05	228	MM	n.a.	1
19	77	m	λ	2.43	1714	MM	PGNMID	4
20	78	m	κ	2.90	1091	MGRS	no pathology	6
21	85	f	λ	0.91	254	MM	n.a.	28
22	73	m	κ	0.97	54	MM	n.a.	2

*LC, light chain; λ, lambda light chain; κ, kappa light chain; FGN, monotypic fibrillary glomerulonephritis; LCPT, light-chain proximal tubulopathy; MGRS, monoclonal gammopathy with renal significance; MGUS, monoclonal gammopathy with undefined significance; MM, multiple myeloma; n.a., not available; PGNMID, proliferative glomerulonephritis and monoclonal immunoglobulin deposits; U[P/C], urinary protein/creatinine ratio given in mg/g; sCr, serum creatinine given in mg/dL after 1-year follow-up. Disease duration is given in years*.

#### Patient With LC Kappa

The male patient had a fine-needle biopsy of the kidney, which showed multiple tubular casts with immunohistochemical reactivity to kappa LC–specific antibodies, conversely negative for lambda LCs. Peritubular inflammatory reaction with mononuclear leukocyte infiltration and interstitial fibrosis, as well as atrophy of tubular cells, was characterized. Glomerular structure was without evidence of pathology; especially no LC deposits were present. This was confirmed by electron microscopy showing no evidence of fibrillary deposit structures.

#### Patient With LC Lambda

The male patient with lambda LC had a bone biopsy performed, but no kidney biopsy due to a deranged coagulation status and poor general condition. In urinary sediment cytoslides, a remarkable number of LC casts (five casts per optical field) were visualized. The individual LC casts were collected under microscope observation and were subjected to mass spectrometry. Results of these date were published recently (16).

### Urine Collection

Clean-catch urine was collected in sterile containers and immediately centrifuged at 3,000 revolutions/min (RPM) for 10 min. Precleared urine was frozen in 3.5-mL aliquots at −80°C for further analysis.

### LC Purification

Precleared urine was treated with saturated ammonium sulfate solution. In brief, urine was mixed with equal volume of saturated ammonium sulfate solution at room temperature. The resultant mixture turned opaque and was transferred into ultracentrifuge tubes (polycarbonate, Prod# 343778; Beckman Coulter) and fitted into the TLA120.2 rotor of an Optima™ MAX-XP ultracentrifuge. Following 1-h centrifugation at 40,000 RPM = 69,000*g*, the supernatant was separated from the pellet. The pellet was redissolved in 1/2 phosphate-buffered saline (PBS) immediately. The redissolved protein was loaded onto a sodium dodecyl sulfate–polyacrylamide gel electrophoresis (SDS-PAGE) and run under non-reducing conditions using TRIS–glycine–SDS as running buffer. The resultant gel was stained by Coomassie blue, followed by destaining in order to visualize LC oligomeric or monomeric structures.

The recovered LC protein was dialyzed in slide dialyzers (Slide-A-Lyzer Dialysis Cassette, Prod# 66330, Thermo Scientific) against PBS at 4°C for 48 h with two changes of the PBS dialysis buffer. These LCs were subsequently used for protein array screening.

### LC Binding to Renal Proteins Immobilized on ELISA Plates

Two hundred milligrams of human renal tissue was homogenized in 1 × tissue lysis buffer (Prod# 9803, Cell Signaling) containing protease inhibitors (cOmplete tablets, Mini EASYpack, Prod# 04693124001, Roche). The tissue lysis was carried out in a Precellys 24 lysis and homogenization machine. Precleared lysate was diluted in PBS (1:1) containing protease inhibitors such as above, and 100 μL was applied to each well of a 96-well flat-bottomed ELISA plate for coating at 4°C overnight. Following a blocking procedure with 1 × blocking solution (Prod# 50-61-01, KPL) for 1 h, urine samples were prepared. Urine samples were diluted 1:6 in PBS and incubated at 37°C for 1 h. ELISA plate washing was carried out with tween phosphate buffered saline (TPBS) on an automated ELISA washing machine applying 300-μL wash solution to each well in three cycles. For development of LC/immunoglobulin binding, rabbit anti-human immunoglobulin and LC-specific antibody (PO212, Dako), diluted 1:1,250 in 1 × RayBiotech buffer (EL-ITEME2), was incubated for 1 h at 37°C. Following the second washing procedure using TPBS and the automated ELISA washing machine, the LC/immunoglobulin binding was developed using the dual-component peroxidase substrate solution (Prod# 50-65-00 and 50-76-01, KPL). The resultant signal was stopped by adding 50 μL of 2N HCl, and signals were quantitated by an ELISA reader at 450 nm. Sample and control measurements were carried out in triplicate.

### Protein Array Screening

Human protein microarrays (HuProt™, Human Proteome Microarrays, Cambridge Protein Arrays) were screened at 4°C. Arrays were blocked in 5% human serum albumin (wt/vol) in Tris-buffered saline, 0.1% Tween (TBST), for 60 min followed by incubation with either purified LC protein at 1 μM for 1.5 h. Samples were applied to the microarray surface and a coverslip placed on the sample. After incubation, the microarray was washed for 5 × 2 min in TBST. Alexa Fluor 642 polyclonal goat anti-human heavy chain– and LC-specific secondary antibody (A21445, Invitrogen), diluted in 5% human serum albumin, was then incubated on the microarray at a concentration of 1 μg/mL for 60 min prior to further washing for 5 × 2 min in TBST, rinsing in deionized water, and drying by spinning in a centrifuge at 250*g* for 3 min. The microarrays were imaged on a Genepix 4000B scanner (Axon Instruments). The PMT gain settings were set at 450 for the 635-nm laser with a focus position of 10 μm. A lot-specific gal-file was used to develop.gpr result files from the array scans, and these files were analyzed with a software script developed in house.

### Software and Statistical Analysis of Microarray Data

A python script called MicroarraySF was written to statistically analyze the .gpr files. The resulting files contained the calculated total fluorescence (the F635 median minus the background from the B635 median), as well as the *Z* scores for each protein. A signal-to-noise ratio cutoff of two was imposed, and a *Z* score cutoff of three was used, as reported previously (17). Flagged proteins with a value of <0 were also filtered out. The output file contained only the proteins that met the parameters and gave new statistical information such as the *Z* scores calculated from the F635 median value.

Data management and data analysis were conducted by GraphPad Prism (GraphPad Prism version 7.00 for Windows, GraphPad Software), as well as Microsoft Excel (Microsoft). A linear regression model was deployed to analyze the association between LC binding intensity and serum creatinine as renal function parameter at 1-year follow-up. The regression coefficient is reported with 95% confidence intervals, and a two-sided *p* < 0.05 was considered significant.

### Preparation of LC Affinity Columns and Renal Protein Affinity Chromatography

The redissolved LCs were dialyzed against PBS at 4°C for 3 days applying three changes of dialysis fluid (PBS). Following swelling and washing CNBr-activated Sepharose 4B with 1 mM HCl, the LC protein was mixed with 1.5 mL gel in stopped disposable column container and rotated for 1 h at room temperature. The column was washed with PBS buffer and exposed to 1 M ethanolamine, pH 8.0, for 2 h. Following the blocking procedure, the column was mock eluted with elution buffer (50 mM glycine, 0.15 M NaCl, 0.1% Tween 20, pH 2.7). Before protein binding, the column was washed with PBS.

Renal whole-cell lysate was precleared by centrifugation at 13,000*g* for 10 min at 4°C, filtered through a 0.22-μm filter, and loaded onto the columns, followed by rotation of the column devices at room temperature for 1 h. The column was washed with 10 column volumes of PBS followed by 10 column volumes of TPBS and then eluted with elution buffer as indicated above in a stepwise mode using 100 μL for collecting in separated tubes.

Twenty microliters of each fraction was then loaded onto a 12% SDS-PAGE gel and run under denaturing conditions. Following the entrance of the loaded protein by 1 cm into the resolving gel, the electrophoresis was stopped, and the gel was stained by Coomassie blue. After destaining, the protein-containing parts of the lane were cut out of each gel separately for kappa and lambda and submitted to proteomics digestion and peptide mass spectrometry.

### Peptide Mass Spectrometry

The procedure was essentially carried out as indicated in earlier work (16). In brief, following trypsin digestion, peptides were further cleaned using a C18 column and then injected into the UltiMate 3000 RSLC nano HPLC (Thermo Fisher Scientific). This HPLC system is linked to a Q Exactive HF mass spectrometer (Thermo Fisher Scientific) using a Proxeon Nanospray source (Thermo Fisher Scientific).

## Results

Multiple myeloma is a common cause for various renal diseases, and a high percentage of affected patients show signs of renal impairment (1, 18). For this reason, patients with monoclonal gammopathies were selected from the myeloma outpatient clinic who showed different extents of protein excretion, with some of them showing signs of renal impairment. Demographic data of study patients are given in Table 1.

**Figure 1:**
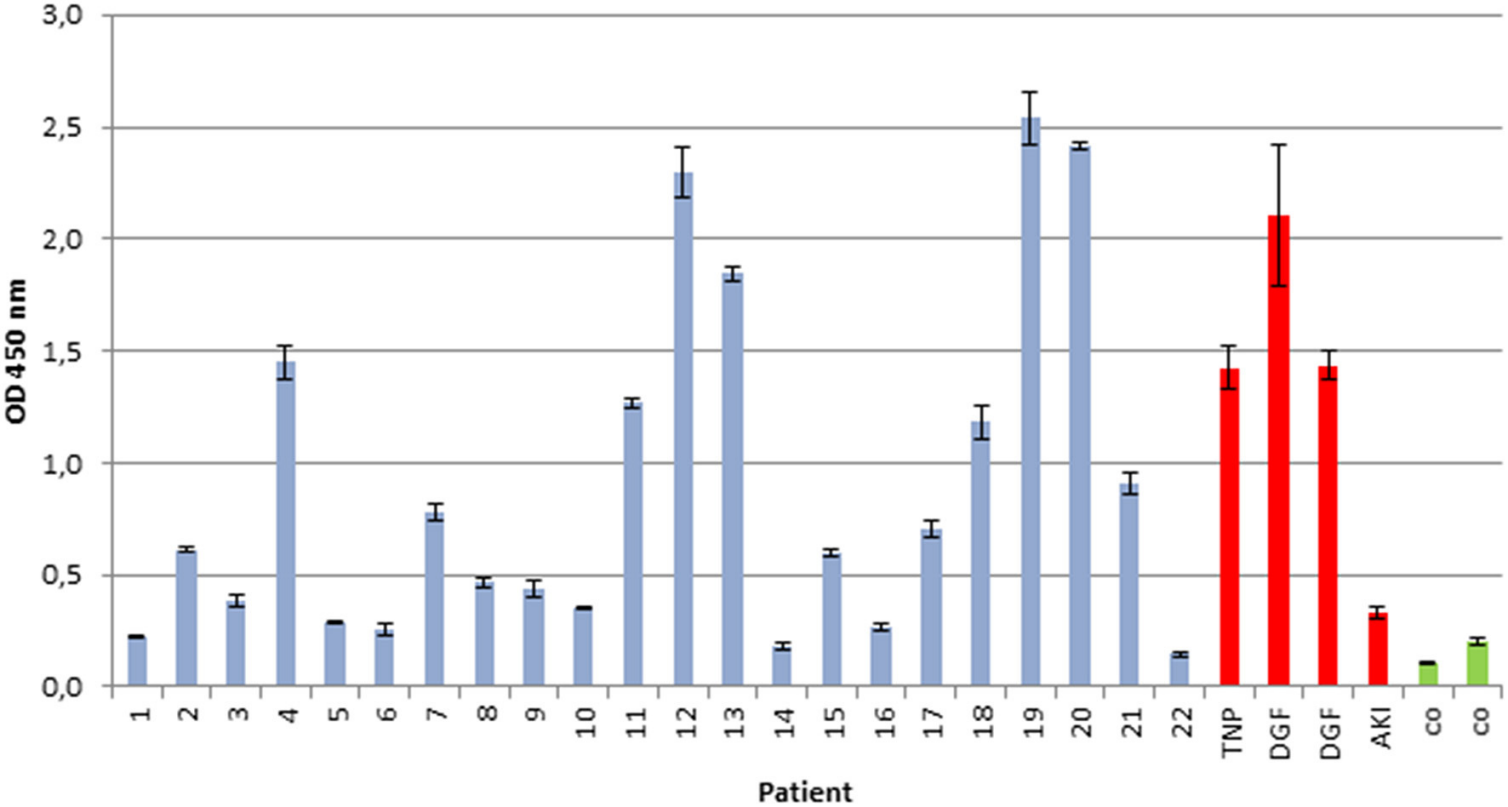
Immunoglobulin/LC binding to immobilized renal whole-cell lysate. Urine of 22 patients with multiple myeloma and plasma cell dyscrasia was incubated in ELISA plates, coated with whole-cell renal lysate, and compared with urine of four patients with renal reperfusion injury (DGF, delayed graft function, red), transplant nephropathy (TNP, red), acute kidney injury (AKI, red), and two healthy controls (CO, green). All experiments were carried out in triplicates. Demographics of patients with multiple myeloma or plasma cell dyscrasia are given in Table 1.

We examined whether the secreted myeloma protein LCs would bind to immobilized renal whole-cell protein on ELISA plates. As depicted in Figure 1, the extent of LC/immunoglobulin binding of patients with multiple myeloma or plasma cell dyscrasia was evaluated and compared with control patients (patients with ischemic–reperfusion injury following renal transplantation as well as healthy subjects). The extent of LC/immunoglobulin binding to renal whole-cell lysates varied among multiple myeloma patients and was found similar to the extent of patients suffering from renal reperfusion injury who also secrete LCs and immunoglobulins due to damage of the glomerular filtration barrier components.

**Figure 2:**
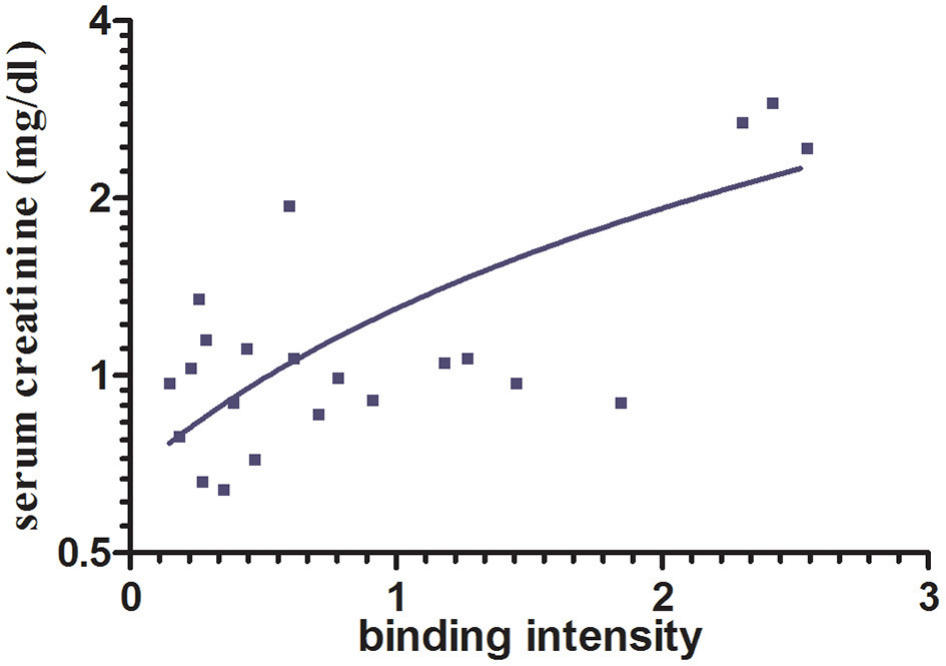
Association between immunoglobulin/LC binding to renal whole-cell lysate by ELISA and serum creatinine after 1-year follow-up. A significant association between immunoglobulin/LC binding intensity and the serum creatinine after 12 months (*p* < 0.0001, *n* = 22) was shown. Binding intensity was measured by photometrical density (OD 450 nm).

To analyze whether the intensity of *in vitro* immunoglobulin/LC binding would allow prediction of the impact on progressive renal dysfunction, a linear regression analysis was performed. Hereby, a significant association between LC binding intensity to renal whole-cell lysate in the ELISA, measured by photometrical density at OD 450 nm, and serum creatinine after a 1-year follow-up was detected, *R* = 0.737 (95% confidence interval, 0.457–0.884; *p* = 0.0001) (Figure 2).

**Figure 3:**
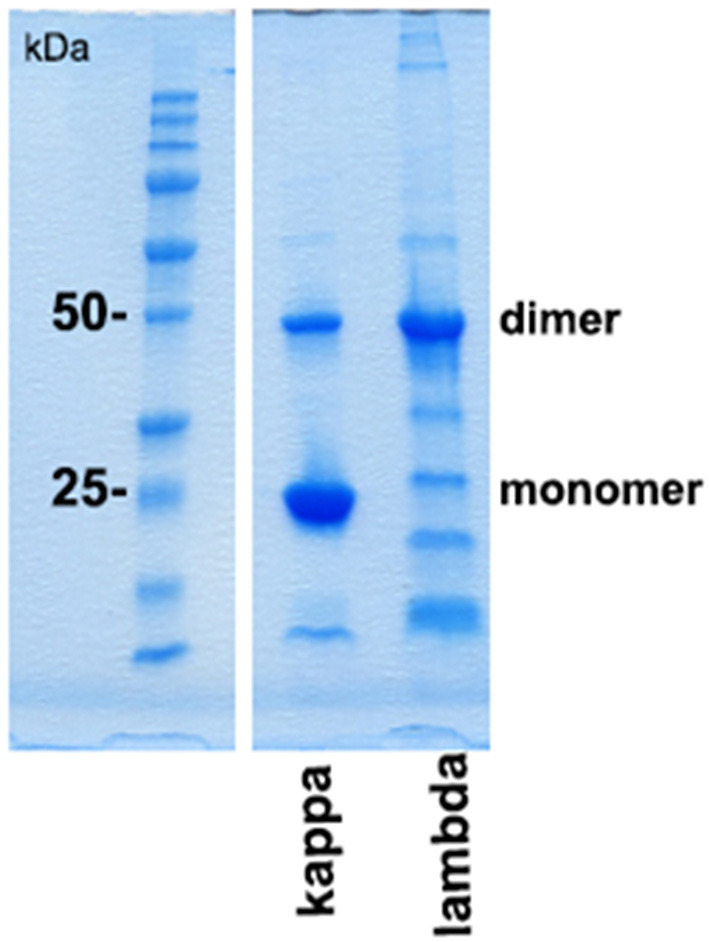
SDS-PAGE gel analysis of purified urine light chains kappa and lambda. Dimers and monomers as well as fragments of LCs are indicated at the right lane. The protein molecular weight marker is indicated on the left lane separated from the other part of the gel. The gel was stained by Coomassie blue following electrophoresis using TRIS-glycine-SDS running buffer. This experiment represents one out of two.

Protein folding as well as interprotein binding and aggregation is assumed to be involved in the pathomechanism of cast formation, intracellular LC deposition, and amyloid formation in paraprotein-associated renal disease. Of particular interest is cast nephropathy, as it represents the most frequently observed manifestation of such disorder in multiple myeloma (1). Therefore, the monoclonal LC kappa and lambda were purified from urine of patients presenting with *acute kidney injury* due to cast nephropathy. Its grade of purification was visualized by SDS-PAGE (Figure 3).

Using these purified LCs, the protein array HuProt™ was screened with equal concentration of lambda and kappa protein each on separate arrays. As demonstrated in Supplementary Figure 1, the lambda LC binding signals were approximately 40% higher at most of the significant interaction partner proteins when compared with kappa signals.

**Figure 4:**
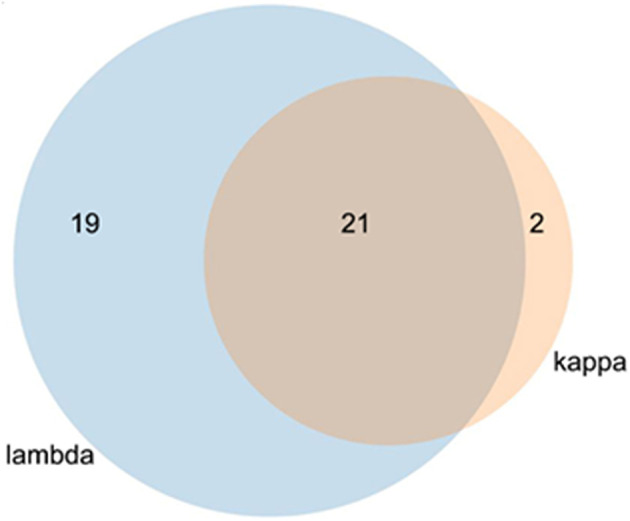
Venn diagram of kappa and lambda overlapping stains. The lambda light chain interacted with 40 individual proteins and the kappa LC with 23 proteins. Of the total of 42 proteins, 21 interacted with both LCs.

In the protein array, a total of 40 interaction proteins were identified in lambda screens. The number of kappa LC interacting proteins was lower (in total 23), whereas 21 interactors were binding to both lambda and kappa LCs (Figure 4, Tables 2, 3).

**Table 2:** Localization and expression of proteins interacting with both kappa and lambda light chain.

**Cell surface protein**	**Intracellular protein**	**Renal expression score**	**Nephron tubule expression score**	**Interacting light chain**
C1QTNF2		64.79	n.a.	λ and κ
	CCNG1	99.18	99.48	λ and κ
CYAT1		n.a.	n.a.	λ and κ
DIXDC1		85.59	81.60	λ and κ
	FAM160B2	97.55	62.70	λ and κ
	GDPD5	79.12	n.a.	λ and κ
	KCNAB1	72.78	53.25	λ and κ
LPAR4		84.89	n.a.	λ and κ
	PARS2	78.19	n.a.	λ and κ
	PCSK7	89.41	74.10	λ and κ
	PPP2R5D	91.03	78.49	λ and κ
	QDPR	98.49	89.84	λ and κ
	RNF7	97.33	91.09	λ and κ
	SCLT1	92.75	n.a.	λ and κ
SIRPB1		71.03	n.a.	λ and κ
	SNX33	84.09	n.a.	λ and κ
TMEM106B		98.45	90.42	λ and κ
TMEM116		90.93	n.a.	λ and κ
TRGC1		68.89	n.a.	λ and κ
	VRK2	86.66	73.99	λ and κ
	ZADH2	89.07	n.a.	λ and κ

*Kappa and lambda LC interaction proteins were categorized for membrane domain containing cell surface or intracellular/cytoplasmic localization (according to the Human Protein Atlas and UniProt database). Renal expression was ascertained and the expression score for adult kidney as well as nephron tubule was extracted from the Bgee database (n.a. = data not available). Detailed protein names are listed in Supplementary Table 1*.

**Table 3:** Localization and expression of interacting proteins unique to either lambda or kappa light chain.

**Cell surface protein**	**Intracellular protein**	**Renal expression score**	**Nephron tubule expression score**	**Interacting light chain**
AQP5		70.48	n.a.	λ
	CLIP4	85.19	n.a.	λ
	COX15	92.96	89.43	λ
	FAM127B	96.03	73.64	λ
	GARS	96.88	93.36	λ
IL12RB1		66.12	n.a.	λ
	KCNAB2	94.79	83.70	λ
	MECR	92.97	75.24	λ
	PRH1	72.97	n.a.	λ
	RBM47	99.70	98.71	λ
	RPRD1A	92.19	80.74	λ
	TLK1	95.59	91.95	λ
	TRIM21	83.33	n.a.	λ
	VDR	90.64	93.84	λ
	WIPF1	89.61	83.53	λ
ZDHHC5		94.03	n.a.	λ
	ALB	98.91	99.79	λ
	CRYZ	99.26	99.22	λ
ECHDC1		97.28	91.70	λ
	BAIAP2L1	84.89	n.a.	κ
	CDK10	98.99	85.38	κ

*The upper part of the table demonstrates lambda specific, the lower part kappa specific binding partners*.

*Kappa and lambda LC interaction proteins were categorized for membrane domain containing cell surface or intracellular/cytoplasmic localization (according to the Human Protein Atlas and UniProt database). Renal expression was ascertained and the expression score for adult kidney as well as nephron tubule was extracted from the Bgee database (n.a. = data not available). Detailed protein names are listed in Supplementary Table 1*.

In order to evaluate the potential disease relevance of LC interactors, their subcellular localization and renal expression were verified by database mining such as the Human Protein Atlas and UniProt. The numeric kidney and nephron tubule expression extent was extracted from the Bgee database (Tables 2, 3). Eight of the kappa and lambda interactors were surface proteins composed of transmembrane domains and verified cell surface structures.

A short functional description of each of the proteins interacting with the two LC subtypes kappa and lambda and a statement about their potential physiological function and involvement in the pathology of tubular epithelial cells is given in Supplementary Data 1.

### Lambda Binding Partners

Of the HuProt™ array that identified 42 LC binding proteins, 19 proteins were found to solely bind to the lambda type LC. Four of them were cell surface proteins (first column of Table 3), with two (ZDHHC5, ECHDC1) being highly expressed in the kidney (expression score >94). Of particular note is the voltage-gated potassium channel subunit beta-2 (KCNAB2), a plasma membrane protein also highly expressed in the kidney (expression score >94) that functions at the cytoplasmic side of cell surface channels and is involved in ion transport.

### Kappa Binding Partners

Only two of the 42 binding partners recognized by the HuProt™ array were kappa LC–specific interactors. Of note here is the cyclin-dependent kinase 10 (CDK10) that is highly expressed in the kidney (expression score >98) and that is involved in cell cycle–dependent processes such as tubular cell regeneration, a constantly ongoing mechanism in mammalian nephrons.

### Protein Confirmation

Cubilin and megalin binding to LC and their uptake at the proximal tubule has been shown in previous studies (11, 19). As we could not show cubilin and megalin as interaction molecules in the HuProt™ array screening, we assumed that the recombinantly generated proteins spotted on the array did not represent the full spectrum of the *in vivo* tertiary and quaternary structure of the proteins. Therefore, we designed a LC affinity chromatography and could indeed verify cubilin and megalin (Table 4).

**Table 4:** Detection of megalin and cubilin by mass spectrometry of light chain affinity column eluates.

**Accession**	**Description**	**Genes**	**MW [kDa]**	**Lambda LC**		**Kappa LC**	
				**Norm. area**	**No. Peptides**	**Norm. area**	**No. Peptides**
P98164.3	Megalin	LRP2	521,6	7,59E+05	21	6,03E+05	20
O60494.5	Cubilin	CUBN	398,5	4,13E+05	8	3,73E+05	11

*Abundance of peptides is calculated as area under the curve (norm. area) of highly specific peptide peaks. Several peptides were identified as specific for megalin and cubilin (no. peptides)*.

## Discussion

Renal amyloidosis, monoclonal immunoglobulin deposition disease, LC proximal tubulopathy, cast nephropathy, and crystal storing histiocytosis are complications associated with monoclonal gammopathies and multiple myeloma. Such manifestations are associated with poor clinical outcome when not diagnosed and treated in early stages. Protein folding and LC interaction with cell surface proteins influence the site and type of LC deposition or transformation into fibrils (20). In this study, we searched for proteins that directly interact with LCs at the nephron. In a first step, this was investigated by an ELISA method using immobilized renal whole-cell protein and patients' urine. Urine immunoglobulin/LC binding could be verified and was much higher in patients who showed progressive renal failure when analyzed 1 year later by renal function parameters. In a second step, kappa and lambda LCs were purified each from a different patient with myeloma, and protein microarrays were screened for identification of potential binding partners for kappa and lambda LC. For both kappa and lambda LC binders, 21 different proteins expressed by renal cells were identified, all of them involved in renal cell activity. Of particular note is the SCLT1 protein named the sodium channel and clathrin linker 1 (21), which might act as a member of a potential transport mechanism by which the LCs are geared to the coated pits and endosomes, which later fuse with lysosomes (20). The SCLT1 is also named CAP-1 and regulates the Na(v)1.8 channel density at the cell surface, and the lysosome has been attributed a specific site for fibrillogenesis in a mouse model of LC amyloidosis (20). More interestingly, the TMEM106B protein is involved in lysosome trafficking and formation (22) and might therefore, when disturbed and partly inhibited by abundant LC presence, represent another cornerstone in LC-induced degenerative tubular nephropathy. This might also be of relevance in the mesangial transformation (23) and fibrillogenesis, which has been studied before (20). A second point of note is the KCNAB1 and KCNAB2 channel proteins involved in potassium transport. Whether the blockage through LCs can cause the acquired Fanconi syndrome has to be left open, the relevance for paraprotein associated neuropathies is thereby more likely, and this topic deserves further research. Proteins that bind to both lambda and kappa are specifically described in Supplementary Data 1, both in terms of their physiological function and their potential pathomechanism in renal cells when partially inhibited or blocked by interaction with an overwhelming amount of monoclonal LC/immunoglobulin.

An interesting observation of our study is that the LCs did not bind in a direct mode to the spotted cubilin or megalin on the array. The manufactured proteins for array printing might be linearized and not glycosylated. Either a specific tertiary structure or an interaction mediator should be of relevance, because according to the previous literature these two proteins ought to represent the internalizing factors (19). However, we confirmed in LC-Sepharose affinity chromatography the binding of cubilin and megalin to both LC types kappa and lambda in almost similar quantity.

This work demonstrates the impact of recently developed tools and methods of protein analysis including microarray screening to screen for a broader range of interaction partners. Whether these newly identified interactors might be involved in cellular uptake could not be researched by these methods, but some of them including SIRPB1, VRK2, ZADH2, and others are certainly involved in signal transduction and initiation of proinflammatory processes at the tubular structures. The intracellular binding partners identified in this study might be relevant in LC protein internalization, accumulation, intracellular transport, and its way to initiate a redox signaling (24) and transformation of mesangial cells (23). In this line of thought, the seven-transmembrane spanning receptor protein LPAR4, binding to both LCs kappa and lambda, might be involved in cell activation. It has been demonstrated earlier that nuclear factor κB might be activated following LC endocytosis (24). Our screens now demonstrate that activation could already be initiated by binding to cell surface proteins via the tubular cell brush border.

The broad spectrum of interaction partners explains the notion that a high LC concentration in urine is associated with more than a single pathological entity in the kidney. The different interaction capabilities depending on the subtype of LC can influence the pathomorphological features. In this respect, intracellular interactors might be of significant relevance. Our data showing higher lambda binding levels might also reflect clinical observations that the lambda paraprotein is potentially more harmful and more likely to cause clinicopathological changes than kappa paraproteins.

The main limitation of this study is that as recombinant proteins are spotted at the HuProt™ array, only primary structure-related protein–protein interactions can be detected. Therefore, protein interactions due to tertiary and quaternary structures may not be identified, which might be a determinant factor for LC interaction *in vivo*. In our study, this applies, for example, to the two well-described transport proteins of LCs cubilin and megalin, which we could not detect by the HuProt™ array, although we could detect them by LC affinity chromatography and subsequent proteomic identification of eluted binders. However, the proteomic workup of LCs goes beyond the scope of this study and is the focus of a follow-up.

Continuous advances in protein analysis, applied to clinically relevant questions, provide detailed insight into disease-causing mechanisms. The results of our study indicate 42 cellular LC binding partners with potential pathomechanical relevance that may contribute to the induction and progression of LC-associated diseases and kidney injuries. This provides new perspectives for targeted diagnostic and therapeutic measures in the future.

## Data Availability Statement

Protein data were downloaded from the Human Protein Atlas (https://www.proteinatlas.org), UniProt (https://www.uniprot.org/) and Bgee (https://bgee.org) databases and according accession numbers are provided in Supplementary Table 1.

## Ethics Statement

The studies involving human participants were reviewed and approved by Ethics committee of the Medical University of Vienna, Austria. The patients/participants provided their written informed consent to participate in this study.

## Author Contributions

TR, SP, LW, and WW conceived and designed the study. LW and CL did the statistical analysis. DO'C, CL, SK, HA, AS, and LW analyzed and interpreted the data. TR, SP, DO'C, CL, SK, HA, AS, LW, NL, and WW critically revised the manuscript for important intellectual content. All authors contributed to the article and approved the submitted version.

## Conflict of Interest

The authors declare that the research was conducted in the absence of any commercial or financial relationships that could be construed as a potential conflict of interest.

## Supplementary Material

Supplementary Figure 1 Proto Array HuProt™ screening using purified human kappa and lambda light chain. Alexa Fluo 642 polyclonal goat anti human heavy and light chain specific secondary antibody was applied as detection reagent. The blue line indicates the staining intensity of lambda and the red line of kappa for the 400 most significant proteins signals.
