# Supplementary material for: Renal Expression of Light Chain Binding Proteins
Source: Front Med (Lausanne). 2021 Jan 13;7:609582. doi: 10.3389/fmed.2020.609582 (PMC7838590; doi:10.3389/fmed.2020.609582)
Supplement: Supplementary file 1 [file Table_1.docx]

**Supplementary Table 1.**

**Interacting proteins common to both (lambda and kappa) purified light chain array screens**

*Indicates cell surface expression

**C1QTNF2:** Complement C1q tumor necrosis factor-related protein 2*

**CCNG1**: Cyclin-G1

**CYAT1:** Immunoglobulin lambda light chain-like*

**DIXDC1:** Dixin*

**FAM160B2:** Protein FAM160B2

**GDPD5:** Glycerophosphodiester phosphodiesterase domain-containing protein 5

**KCNAB1:** Voltage-gated potassium channel subunit beta-1

**LPAR4:** Lysophosphatidic acid receptor 4*

**PARS2:** Probable proline--tRNA ligase, mitochondrial

**PCSK7:** Proprotein convertase subtilisin/kexin type 7

**PPP2R5D:** Serine/threonine-protein phosphatase 2A 56 kDa reg. subunit delta isoform

**QDPR:** Dihydropteridine reductase

**RNF7:** RING-box protein 2

**SCLT1:** Sodium channel and clathrin linker 1

**SIRPB1:** Signal-regulatory protein beta-1*

**SNX33:** Sorting nexin-33

**TMEM106B:** Transmembrane protein 106B*

**TMEM116:** Transmembrane protein 116*

**TRGC1:** T cell receptor gamma constant 1*

**VRK2:** Serine/threonine-protein kinase VRK2

**ZADH2:** Prostaglandin reductase-3

**Interacting proteins unique to lambda light chain array screen**

*Indicates cell surface expression

**AQP5:** Aquaporin-5*

**CLIP4:** CAP-Gly domain-containing linker protein family member 4

**COX15:** Cytochrome c oxidase assembly protein COX15 homolog

**FAM127B:** Retrotransposon Gag-like protein 8A

**GARS:** Glycyl-tRNA synthetase

**IL12RB1:** Interleukin-12 receptor subunit beta-1*

**KCNAB2:** Voltage-gated potassium channel subunit beta-2

**MECR:** Enoyl-[acyl-carrier-protein] reductase, mitochondrial

**PRH1:** Salivary acidic proline-rich phosphoprotein 1/2

**RBM47:** RNA-binding protein 47

**RPRD1A:** Regulation of nuclear pre-mRNA domain-containing protein 1A

**TLK1:** Serine/threonine-protein kinase tousled-like 1

**TRIM21:** E3 ubiquitin-protein ligase TRIM21

**VDR:** Vitamin D3 receptor

**WIPF1:** WAS/WASL-interacting protein family member 1

**ZDHHC5:** Palmitoyltransferase ZDHHC5*

**ALB:** Serum Albumin

**CRYZ:** Quinone oxidoreductase

**ECHDC1:** Ethylmalonyl-CoA decarboxylase*

**Interacting proteins unique to kappa light chain array screen**

**BAIAP2L1:** Brain-specific angiogenesis inhibitor 1-associated protein 2-like protein 1

**CDK10:** Cyclin-dependent kinase 10
