# Supplementary figures and images for: Renal Expression of Light Chain Binding Proteins
Source: Front Med (Lausanne). 2021 Jan 13;7:609582. doi: 10.3389/fmed.2020.609582 (PMC7838590; doi:10.3389/fmed.2020.609582)

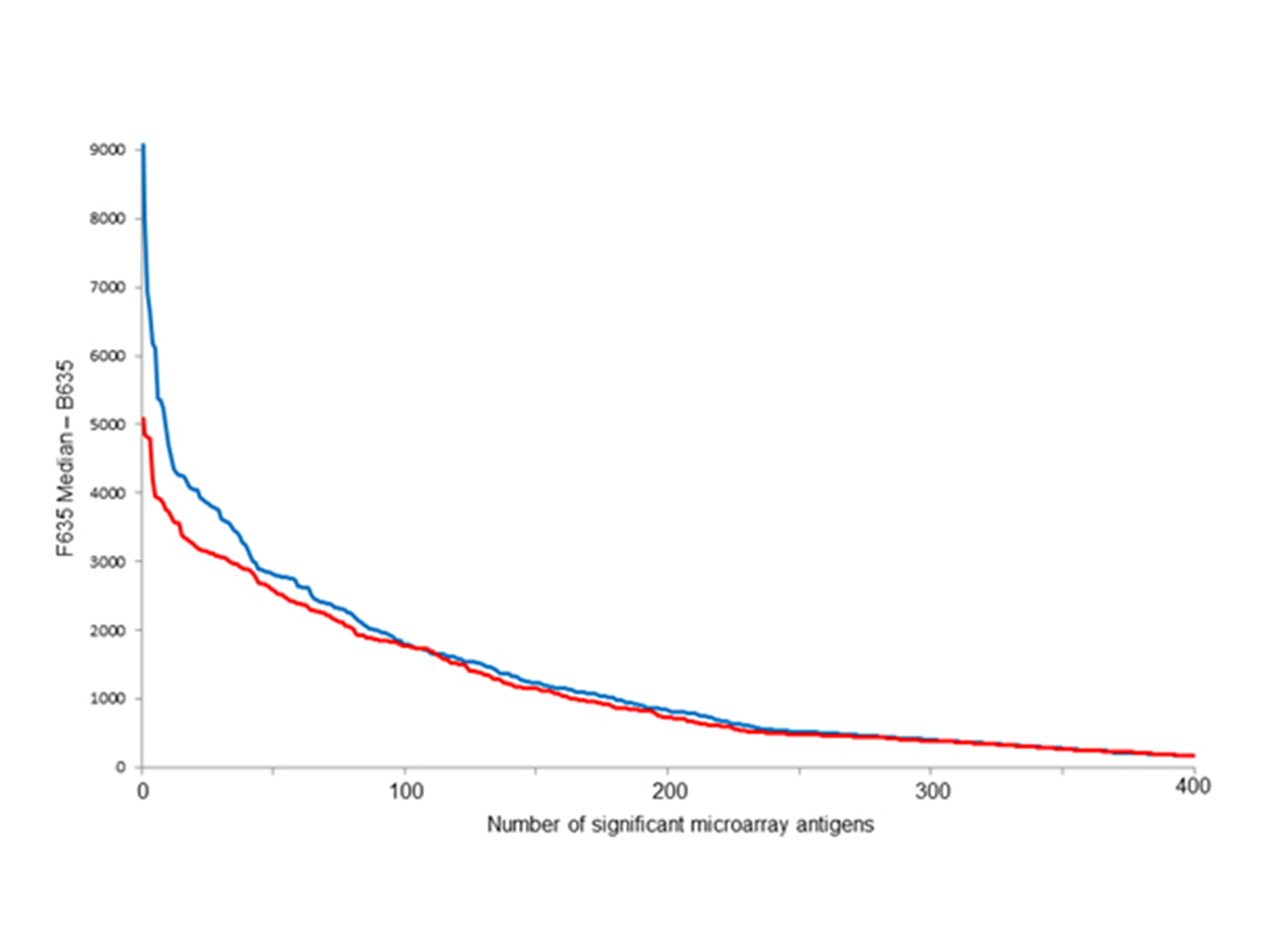

Supplement: Supplementary Figure 1 — Proto Array HuProt™ screening using purified human kappa and lambda light chain. Alexa Fluo 642 polyclonal goat anti human heavy and light chain specific secondary antibody was applied as detection reagent. The blue line indicates the staining intensity of lambda and the red line of kappa for the 400 most significant proteins signals. [file Image_1.TIF]
